# Small-Lungworm (Protostrongylidae) Infections in Relation to Meat Sheep Breeds, Mediterranean Climates, and Anthelmintic Regimens

**DOI:** 10.3390/vetsci12050471

**Published:** 2025-05-14

**Authors:** Bourhane Bentounsi, Jacques Cabaret

**Affiliations:** 1 Laboratoire de Parasitologie, Institut des Sciences Vétérinaires, Université Mentouri 1, Constantine, Algeria; bbentounsi@umc.edu.dz; 2 Infectiologie et Santé Publique, INRAE and Université de Tours, F-37380 Nouzilly, France

**Keywords:** *Muellerius*, *Neostrongylus*, *Cystocaulus*, climate, anthelmintics, small lungworms

## Abstract

Protostrongylid nematodes (small lungworms) are very common in Mediterranean sheep and have long been recorded in North Africa. Here, the following four species are found: *Muellerius capillaris*, *Neostrongylus linearis*, *Cystocaulus ocreatus*, and *Protostrongylus rufescens*. Previous risk factors studies for protostrongylids have been conducted in a single farm and therefore have limitations. Sixty-one meat sheep farms in north-eastern Algeria were surveyed for protostrongylid infection and anthelmintic treatment in late autumn/early winter. The climates of the nine regions ranged from subhumid to arid for humidity and from mild to cool for winter temperature. The highest infection, estimated by the number of larvae per gram of faeces (LPG), was found in subhumid and semi-arid climates. The Rembi breed was more infected than the Ouled Djellal or their crosses. LPG decreased with increasing number of treatments. The latter was also associated with an increased percentage of *M. capillaris* and a decrease in species diversity. The anthelmintic regimen (ivermectin, levamisole, and albendazole) directly targets gastrointestinal nematodes and indirectly protostrongylids. The use of effective drugs targeting protostrongylids at key moments may provide more effective control.

## Simple Summary

Small lungworms are highly prevalent in Mediterranean meat sheep. Four species of these protostrongylid nematodes are usually present, with varying prevalence. They are climate-dependent due to a two-host life cycle involving terrestrial snails and slugs and sheep or goats. They are also susceptible to anthelmintic treatment. Epidemiological studies are based on seasonal evolution within a single flock or farm, and we studied protostrongylid infection in 61 farms under different climates in Algeria, with different sheep breeds and anthelmintic regimens. At the regional level, the climate, sheep breed, and number of anthelmintic treatments each played a role in the intensity of infection and species diversity. These indications can be used to tailor anthelmintic treatments. In fact, the regions are complex to describe, and it was found that breed selection was related to climate and that increasing the number of anthelmintic treatments had a negative effect on protostrongylid diversity.

## 1. Introduction

There are four common species of protostrongylid nematodes found in domestic sheep: *Muellerius capillaris*, *Neostrongylus linearis*, *Cystocaulus ocreatus*, and *Protostrongylus rufescents* [1]. Protostrongylid nematode [2] infection of sheep is highly prevalent in Mediterranean countries [2]. The prevalence of these small lungworms can reach up to 100% of ewes in North Africa [2] or Syria [3]. It has long been recorded in Algeria [4], Morocco [5], and Palestine [6]. The parasites have a two-host life cycle. The intermediate hosts are mainly terrestrial molluscs (gastropods of the families Helicidae, Arionidae, or Limacidae), although most gastropod species can be infected [7]: 180 species which belong to 27 families have been documented. The definitive hosts are sheep and goats. Some differences in the infection of sheep breeds were sometimes found [8], but others have challenged this [9]. The first-stage larvae (L1), which are transported in the faeces of the definitive hosts during wet periods, randomly encounter the intermediate host snail [2]. Climate can affect larval survival [2], transmission to snails and slugs, and their survival. *Muellerius* infection has been documented as being largely predominant in colder and wetter climates. In contrast, other protostrongylid species have also been recorded in most of the Mediterranean sites that have been the subject of study [2]. The excretion of larvae in faeces (expressed in larvae per gram, LPG) depends on the age of the sheep (ewes are more infected than lambs), the season, and the anthelmintic treatments [10,11]. It should be noted that some treatments only temporarily reduce larval shedding [11,12,13]. There are also differences in the efficacy of the different molecules used for treatment [14]. Anthelmintic treatments against digestive tract strongyles are routinely carried out several times a year and may affect LPG levels and species frequencies within the protostrongylid community. The infection of land snails depends on their age, species, and climate [15,16,17,18]. Only one paper related protostrongylid infection of a wild definitive host (bighorn sheep) to climate [19], and there was also one related to sheep [20]. The aim of the present paper is to determine the influence of climate and the history of anthelmintic treatment under different Mediterranean climates in domestic sheep from farms in infected areas of Algeria.

## 2. Materials and Methods

### 2.1. Farms and Sampling

**Figure 1:**
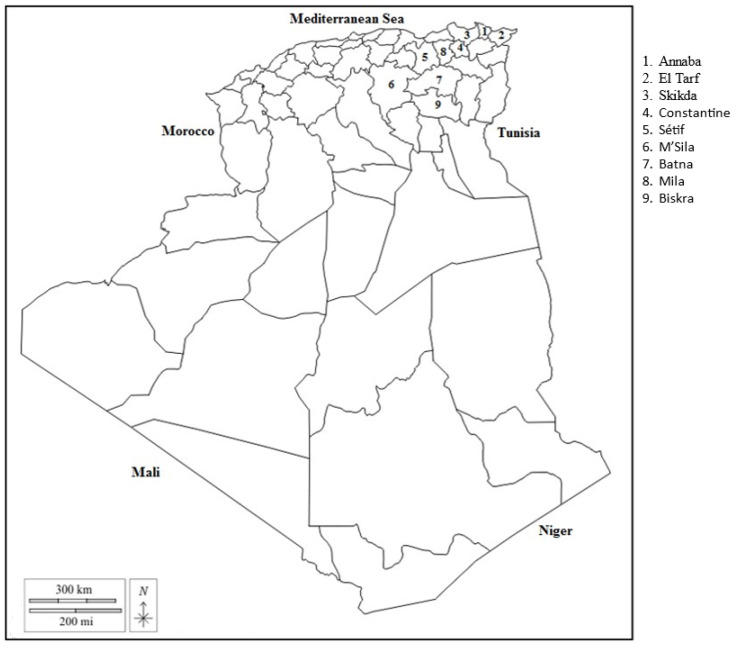
Sampling regions in north-east Algeria.

Sixty-one non-transhumant sheep farms were surveyed from November to January. They were selected to correspond to the different subclimates and willingness of the farmers. Each excursion from Constantine University was carefully planned to coincide with selected villages within the region. The farmers were contacted following the observation of their flocks from the road, and faecal samples were taken only with their express consent. The farms in the coastal area were diversified due to the better soils, with arable farming and mixed livestock (sheep, goats, and cattle) on individual or common pastures. The farms in other areas had extensive sheep farming, with movements on steppe pastures and minimal cereal cultivation. Sheep were kept outdoors all year round. The lambing period was in autumn and spring, so our sampling corresponded to the autumn lambing period. The highest period of larval excretion in sheep was autumn–winter [21]. The farms came from 49 villages in the nine wilayas (administrative regions: Figure 1) of north-east Algeria: Skikda, Annaba, El Tarf, Sétif, Constantine, Mila, M’Sila, Batna, and Biskra. The sheep breeds (Ouled Djellal, Rembi, and their crosses) and the anthelmintics and their history of use were recorded in each farm. Ten random faecal samples of ewes from each farm were processed using Baermann apparatus [22] to extract first-stage larvae. All the larvae collected were identified to species [Supplementary Figure S1], mainly by larval tail morphology. The sampled regions, farms, and climatic data are listed in Table 1. The number of anthelmintic treatments, the class of anthelmintic (albendazole, ivermectin, or levamisole), and the interval between the last treatment and sampling were recorded by questionnaire on 61 farms.

### 2.2. Climate Parameters

The climate in the area under investigation is Mediterranean, but it is also influenced by continental climate and altitude. The coldest month in the area is January and the hottest is July (average 25 °C). Drought lasts from June to September. The climate of the various villages is based on the Emberger classification applied by Côte, 1998 to Algeria. With the construction of the Mila and Setif dams in 2004, there have been some changes in the climate towards higher humidity. The five bioclimates of Emberger [23] are divided into humid, subhumid, semi-arid, sub-arid, and arid. The bioclimates are based on Emberger’s pluviothermic coefficient: its value is positively related to the annual rainfall and negatively related to the annual mean temperature. Côte [24] slightly modified the codification: sub-arid and arid correspond to arid and Saharan climate of Emberger. Subclimates within each bioclimate are defined by the minimum of the coldest month m: hot (m > 7 °C, no frost), mild (3 °C < m < 7 °C, rare frost days), cool (0 °C < m < 3 °C, frequent frost days), and cold (m < 0, frequent deep frost days).

### 2.3. Statistical Analysis

Since the data did not follow a Gaussian distribution, the means were compared using the non-parametric Kruskall and Wallis tests. After a logarithmic transformation to stabilise the variance of the LPG, an analysis of variance (ANOVA) was performed, followed by a post hoc Newman and Keuls test to identify significant differences between means, and a general model of analysis of variance (GLM) when nominal data were included (breed, use of different anthelmintics, etc.). All these statistical analyses were carried out using SPSS 11.5 IBM software. Multivariate analyses (principal component analyses: PCA) on quantitative data and clustering analysis of quantitative and qualitative data (based on the Gower similarity measure [25]) were used to relate a whole set of variables (MVSP 3.1 software [26]). The results of the PCA are presented as graphs with two axes and the relative importance of these axes is expressed in terms of percentage of variance. Each axis is a linear combination of variables constructed to maximise variance; the second axis maximises variance but is also independent of the first axis. The most descriptive variables were located at the periphery of the graph, while the least descriptive were located near the intersection of the axes. The diversity indices were number of species, Shannon [27], and evenness. A correspondence analysis was performed on all variables distributed in two (presence/absence) or more classes to assess the role of each climate, breed, or anthelmintic treatment. It is an adaptation of PCA for categorical data and it analyses a contingency table instead of a correlation/covariance matrix.

## 3. Results

### 3.1. Regional Differences in Infection with Protostrongylids (Table 1)

Significant differences between regions were noted for LPG (non-parametric Kruskall–Wallis, *p* = 0.004). The ANOVA (analysis of variance; *p* = 0.04) on log transformed LPG values showed that the semi-arid with fresh or mild climate (Mila) had the highest protostrongyle infection, and the sub-arid fresh climate (M’sila and Batna) had the lowest. The subhumid regions had an intermediate protostrongylid infection as based on LPG. The best GLM analysis (R^2^ = 0.45) showed that the LPGs were related to region (*p* = 0.004) and the time interval of treatment before sampling (*p* = 0.005). *M. capillaris* LPGs differed significantly (*p* = 0.03) and were mostly found in several subhumid areas (Annaba, El Tarf, and Skikda), whereas they were extremely low in the arid areas. Significant differences were also found for *C. ocreatus* where the most infected regions were the subhumid (Constantine) and semi-arid (Mila) areas. *N. linearis* was significantly more abundant in a semi-arid area (Mila). No significant difference was found for *P. rufescens*, which had a very low prevalence. The regions have different abundant climates, sheep breeds, treatments, and thus further analysis is needed to identify the most significant factors influencing protostrongylid infection.

### 3.2. Climate and Protostrongylid Infection

The LPG of the semi-arid and subhumid climates were not significantly different. The lowest levels of infection were found in sub-arid and arid climates. *M. capillaris* was most common in subhumid mild, *C. ocreatus* in subhumid fresh and semi-arid fresh, and *N. linearis* in semi-arid fresh or mild climates (Table 2).

### 3.3. Sheep Breeds and Protostrongylid Infections

The crossbreeds were found in several subhumid regions (Annaba, El Tarf, Skikda, and Constantine). The Rembi breed was found only in the semi-arid Mila region. The Ouled Djellal were found in four other regions with bioclimates ranging from subhumid to arid. Ouled Dellal and their crosses had a lower LPG than Rembi (*p* = 0.05); this was not due to the number of treatments since it was lower in crosses which were treated less than other breeds (Table 3).

### 3.4. Anthelmintic Practices and Protostrongylid Infections

**Figure 2:**
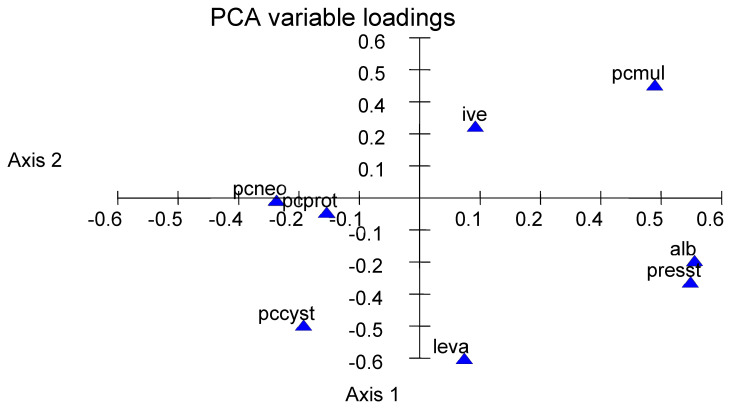
Relationship between the protostrongylid fauna and number of anthelmintic treatments based on principal components analysis (PCA). Codes: alb: albendazole, ive: ivermectin, leva: levamisole, presst: number of treatments per year, pcmul: percentage of *Muellerius capillaris,* pccyst: percentage of *Cystocaulus ocreatus*, pcneo: percentage of *Neostrongylus linearis*, and cprot: percentage of *Protostrongylus rufescens*.

Ivermectin, levamisole, and albendazole were used in 75%, 4%, and 43% of the farms, respectively. The average number of treatments/year was 1.33 and the average time between treatments was 4.88 months. The intensity of treatments (no./year: presst) intended to reduce gastrointestinal nematodes infection, mostly related to the use of albendazole, resulted in a reduction in protostrongylid infection as based on the LPG (Kruskall and Wallis test: *p* = 0.04). The average LPG was 351, 156, and 118 for farmers using zero, one, or two and more anthelmintic treatments per year, respectively. When albendazole was used the percentage of *Muellerius* within the larvae increased from 34 to 51%. Similar conclusions are drawn from the PCA with 40% of inertia with axes 1 and 2 (Figure 2): the pressure of treatment and the use of albendazole are related to an increase in *M*. *capillaris* in proportion (the variables are located on the right of the figure) while the percentage of all other species decreases.

**Figure 3:**
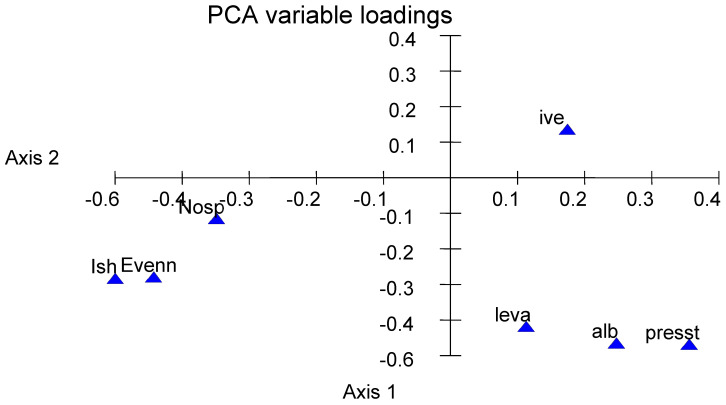
Species diversity in relation to anthelmintic treatments based on principal component analysis (PCA). Codes: Ish: Shannon diversity index, even: evenness, nosp: number of protostrongylid species, alb: albendazole, ive: ivermectin, leva: levamisole, and presst: number of treatments per year.

The PCA relating diversity and anthelmintic treatments had 38% inertia on the first two axes (Figure 3). The diversity of infection (estimated by number and proportion of species, Shannon index IsH, and evenness) decreased as the treatment pressure increased.

### 3.5. A Global Interpretation

**Figure 4:**
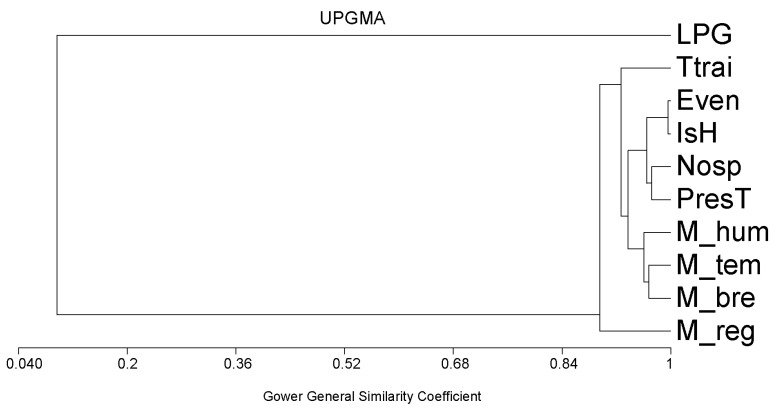
Protostrongylid intensity of infection (LPG) and diversity of species in relation to region, climate, and anthelmintic regimen based on cluster analysis with UPGMA (unweighted pair group method with arithmetic mean). Codes: LPG: larvae per gram of faeces, M_bre: sheep breed, M_hum: bioclimate, M_temp: subtype based on winter temperature, presT: number of treatments per year, nosp: number of protostrongylid species, Ish: Shannon index, even: evenness, and Ttrai: time to last treatment.

Many factors (quantitative or qualitative) interact with protostrongylid infection, and a cluster analysis was performed to understand the relationship between the regions, breeds, climates, and anthelmintic treatments (Figure 4). The qualitative factors were multistate. For example, M_bre corresponded to the three breeds of sheep, coded as one, two, and three. The LPGs were not related to any factor in this analysis. Each region was characterised by a mixture of climatic parameters, sheep breeds, and anthelmintic regimens. (Gower similarity of 0.90, *p* = 0.001). Clearly, the diversity of species was related to anthelmintic regimen (no treatment per year and time to the last treatment). The presence of each sheep breed was highly related to climate parameters.

**Figure 5:**
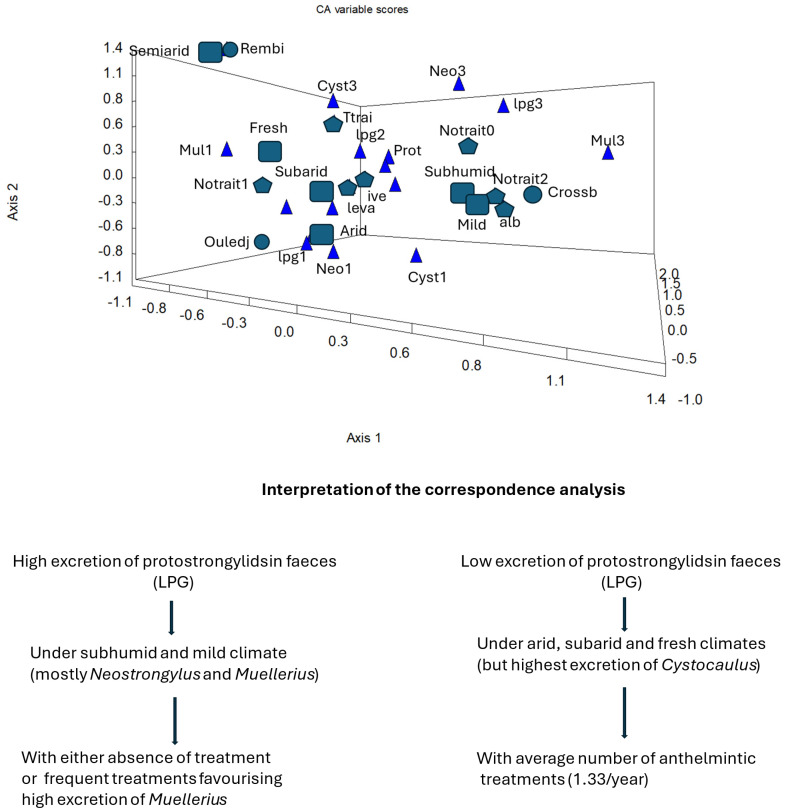
Relationship between protostrongylid infection (lpg), species, anthelmintic treatments, sheep breeds, and climate using correspondence analysis. The variables are: lpg1, the lowest LPG; lpg2, the middle class; and lpg3, the highest number of larvae. Cyst 1: the lowest number of larvae and Cyst 3: the highest, same for *M. capillaris* (Mul 1 and Mul 3) and *C. ocreatus* (Cyst1 and Cyst3). The class 2 of protostrongylid species was not indicated for the sake of clarity. Prot (presence); number of anthelmintic treatments: Notrait 0 (0), Notrait 1 (average), and Notrait 2 (equal to or greater than 2/year). Trait: time to treatment before faecal sampling. ive: ivermectin, alb: albendazole, and leva: levamisole; climates: subhumid, semi-arid, sub-arid, and arid, with a mild and fresh winter; sheep breeds: Ouled Djellal, Rembi, and crossbreeds.

Since each region is a mixed variable with its own climate, adapted sheep breed, anthelminthic treatment regimen, and protostrongylid species. A correspondence analysis was undertaken with each variable transformed into classes (Figure 5). The first, second and third axes represented, respectively, 20.6, 17.8, and 12.5 percent of the variance (or inertia). The main differences with the previous analysis are as follows: (i) climate (humidity and temperature) is a major factor in protostrongylid infection intensity, (ii) it partly determines the proportions of species, (iii) the increasing number of anthelmintic treatments mainly favours *M. capillaris*, and (iv) each sheep breed is found in distinct regions according to climate.

## 4. Discussion

Regions include many characteristics such as sheep breeds, climates, and treatment regimens. They are interesting units because they provide information for control in the area. They indicate when each breed and at what age animals should be treated with anthelmintics [11,14]. The use of strategic treatments with benzimidazoles based on local knowledge of epidemiology has been shown to be effective in Morocco [27]: survival of ewes and their lambs has been significantly improved. We obtained information in several regions of north-eastern Algeria and found that the infection was higher in the subhumid area, as already noted in Spain [20], that the Ouled Djellal breed and its crosses had a lower LPG than the Rembi, and that increasing the number of anthelmintic treatments expectedly reduced the LPG, as noted in Spain [11], Portugal [14], Morocco [28] and Ethiopia [29]. The species proportions of protostrongylids were like another study in north-west Algeria [30] and of those recorded in Morocco [10], with a large predominance of *M. capillaris* and the second most important species being *N. linearis.* However, it differed from the results obtained in Tunisia where *P rufescens* was dominant and *M. capillaris* was the least frequent [31]. This difference could be related to differences in climate or anthelmintic usage.

Climate has been considered as an important factor influencing the presence of different species of protostrongylids in small ruminants. For example, Forrester and Little [19] showed that the presence of *Protostrongylus* in bighorn sheep was positively related to rainfall. In domestic sheep in Spain, relative humidity and rainfall together were related to the number of *N. linearis* larvae per gram of faeces; there was also a correlation between temperature and the percentage of sheep passing *M. capillaris* [20]. In another region of Spain, *C. ocreatus* was found to be the most responsive to climatic conditions such as lower temperature and higher humidity [32]. However, these data were collected in a limited area or on a single farm. We also found that *Muellerius, Neostrongylus*, and *Cystocaulus* were found in certain climates on our 61 farms. In fact, protostrongylids can develop under a wide range of climatic conditions. The case of *M. capillaris* is exemplary as it can be found in equatorial climates [33], in desert areas [2], or near arctic regions [34,35]. The importance of climate per se is not as high as had been thought. In this respect, if we look at the characteristics of these farms we can see that the climates are very much related to the sheep breeds (Figure 4), which in turn correspond to different types of management. The influence of the climate could be linked to the infection of terrestrial molluscs [15,16,17,18], which in turn favoured the infection of sheep. However, this was not studied in the present survey.

The most striking parameter for the proportion of protostrongylid species was the anthelmintic treatment regime (number of treatments per year and time since last treatment). Anthelmintic treatments have a lower efficacy against protostrongylids than against gastrointestinal nematodes [2,36]. Even when good efficacy is observed, it is usually transient and the remaining adults gradually resume larval production after treatment with levamisole [12], or ivermectin [13]. Most studies of anthelmintic efficacy are based on larval excretion in faeces, but there is a good correlation between adult worms in the lungs and larvae in faeces [10] and between the reduction after treatment of both adults and larvae in faeces [36]. The number of treatments and the use of albendazole were associated with a higher proportion of *M. capillaris* (Figure 2). Treatment with benzimidazole (fenbendazole) reduced fecundity in all species, but the reduction was less in *M. capillaris* and *N. linearis* than in *C. ocreatus* and *P. rufescens* in a study in Morocco [36]; this was observed at different doses, from the 5 mg/kg body weight (b.w.) used for gastrointestinal strongyles to the higher doses (10 or 15 mg/kg b.w.) recommended for protostrongyles. The treatments were intended for gastrointestinal strongyles in Algeria and should have favoured both *M. capillaris* and *N. linearis*. This is consistent with the high prevalence of *M. capillaris* and *N. linearis* found in our study. Ivermectin or other macrocyclic lactones will be used more frequently in the future and will result in an increased proportion of *M. capillaris*, as this species has been shown to be the most resistant species to treatment [36,37,38]. Finally, it should be noted that the treatments are mostly directed against gastrointestinal infection and not against the protostrongylids at the key moments (e.g., reduction in land snail infection and thus further sheep infection).

A factor-by-factor analysis of risk can be biassed [39,40,41]. For example, differences in infestation by breed are related to the preferential presence of certain breeds in certain regions, mainly for climatic reasons (see correspondence analysis: Figure 5). The same analysis shows that the importance of the different protostrongylid species is modulated by climate and by anthelmintic treatments. It is therefore possible to refine the influence of these treatments: we find that the absence and the large number of treatments in Figure 5 corresponds to high levels of LPG. It is likely that a high infestation may be the result of (i) many ineffective treatments (due to the presence of *M. capillaris* and/or the use of benzimidazoles at too low a dose), or (ii) the absence of treatment in an area favourable to infestation, such as the subhumid climate.

The morphological approach we used is characterised by a significant time investment, both in terms of the collection of samples and the subsequent microscopic identification of protostrongylid species by experts. The latter could be reduced by means of molecular techniques, although the data on sheep protostrongylids are still limited [42,43]. The implementation of these molecular identifications would facilitate the execution of more extensive studies, encompassing a broader array of climates.

## 5. Conclusions

This survey of sheep protostrongylids has shown that the regional level is suitable for finding solutions to control the infection. However, the different characteristics of the regions are complex and a multivariate analysis is needed to disentangle the role of each factor (local climate, sheep breed, and anthelmintic regimen). Much progress could be made in the use of anthelmintics at risk periods with appropriate doses.

## Figures and Tables

**Table 1:** Protostrongylid species prevalences (based on 30 to 90 ewes) and larvae per gramme of faeces (LPG) in nine regions of north-east Algeria.

Region (Number of Villages)	Climate (Number of Farms)		Average LPG per Farm (SD)	Prevalence (LPG) per Region and Climate			
	Humidity	Winter Temperatures		*Muellerius capillaris*	*Cystocaulus ocreatus*	*Neostrongylus linearis*	*Protostrongylus rufescens*
Annaba (6)	Subhumid (7)	Mild	202 (139)	100 (176)	75 (3)	88 (22)	0 (0)
El Tarf (4)	Subhumid (6)	Mild	268 (542)	100 (187)	67 (5)	83 (76)	17 (0.2)
Skikda (6)	Subhumid (7)	Mild	82 (110)	100 (66)	57 (1)	100 (15)	0 (0)
Constantine (5)	Subhumid (3)	Fresh	126 (214)	100 (15)	83 (84)	100 (28)	0 (0)
	Semi-arid (3)	Mild					
Sétif (9)	Subhumid (9)	Fresh	138 (289)	56 (7)	89 (14)	89 (108)	22 (9)
M’Sila (1)	Sub-arid (3)	Fresh	2 (3)	67 (1)	100 (1)	100 (0,4)	100 (0.4)
Batna(5)	Sub-arid (8)	Fresh	9 (9)	20 (1)	80 (5)	90 (2)	20 (0.8)
	Semi-arid (1)	Fresh					
Mila (7)	Semi-arid (5)	Fresh	521 (640)	38 (1)	100 (83)	100 (437)	0 (0)
	Semi-arid (3)	Mild					
Biskra (6)	Arid (5)	Mild	71 (107)	67 (8)	50 (8)	50 (61)	17 (0.6)
	Sub-arid (1)	Fresh					

**Table 2:** Protostrongylid species (average LPG of the farms) in relation to climate in north-east Algeria.

Climate (No of Farms)	*Muellerius capillaris*	*Cystocaulus ocreatus*	*Neostrongylus linearis*	*Protostrongylus rufescens*
Subhumid mild (20)	142a *	3a	35a	0a
Subhumid fresh (12)	12b	48b	101a	7a
Semi-arid fresh (5)	3c	77b	279b	0a
Semi-arid mild (7)	2c	25a	448c	0a−
Sub-arid fresh (12)	1c	7a	18a	1a
Arid mild (5)	1c	1a	1d	1a
Significance (Kruskall and Wallis test)	*p* = 0.001	*p* = 0.01	*p* = 0.002	*p* = 0.19

* The different letters indicate a significant difference within each protostrongylid species in relation to the different climates.

**Table 3:** Protostrongylid species and number of treatments in relation to breed.

Sheep Breed (Number of Farms)	Number of Treatments	*Muellerius capillaris*	*Cystocaulusocreatus*	*Neostrongylus linearis*	*Protostrongylus rufescens*
Ouled Djellal (26)	1.29 a	114 a	21 a	33 a	0 a
Rembi (7)	1.65 a	1 b	83 b	57 a	0 a
Crossbreed (18)	0.89 b	3 b	4 a	437 b	3 b
Significance (Kruskal and Wallis test)	*p* = 0.05	*p*= 0.00	*p* = 0.05	*p* = 0.00	*p* = 0.05

The different letters indicate a significant difference within protostrongylid species and number of treatments in relation to breed.

## Data Availability

The datasets generated and/or analysed during the current study are available from the corresponding author upon reasonable request.

## Supplementary Materials

The following supporting information can be downloaded at: https://www.mdpi.com/article/10.3390/vetsci12050471/s1, Figure S1: Identification of protostrongylid first-stage larvae of sheep.
