# Supplementary material for: Small-Lungworm (Protostrongylidae) Infections in Relation to Meat Sheep Breeds, Mediterranean Climates, and Anthelmintic Regimens
Source: Vet Sci. 2025 May 14;12(5):471. doi: 10.3390/vetsci12050471 (PMC12115696; doi:10.3390/vetsci12050471)
Supplement: Supplementary file 1 [file vetsci-12-00471-s001.zip › vetsci-3604456-supplementary.pptx]

## Slide 1
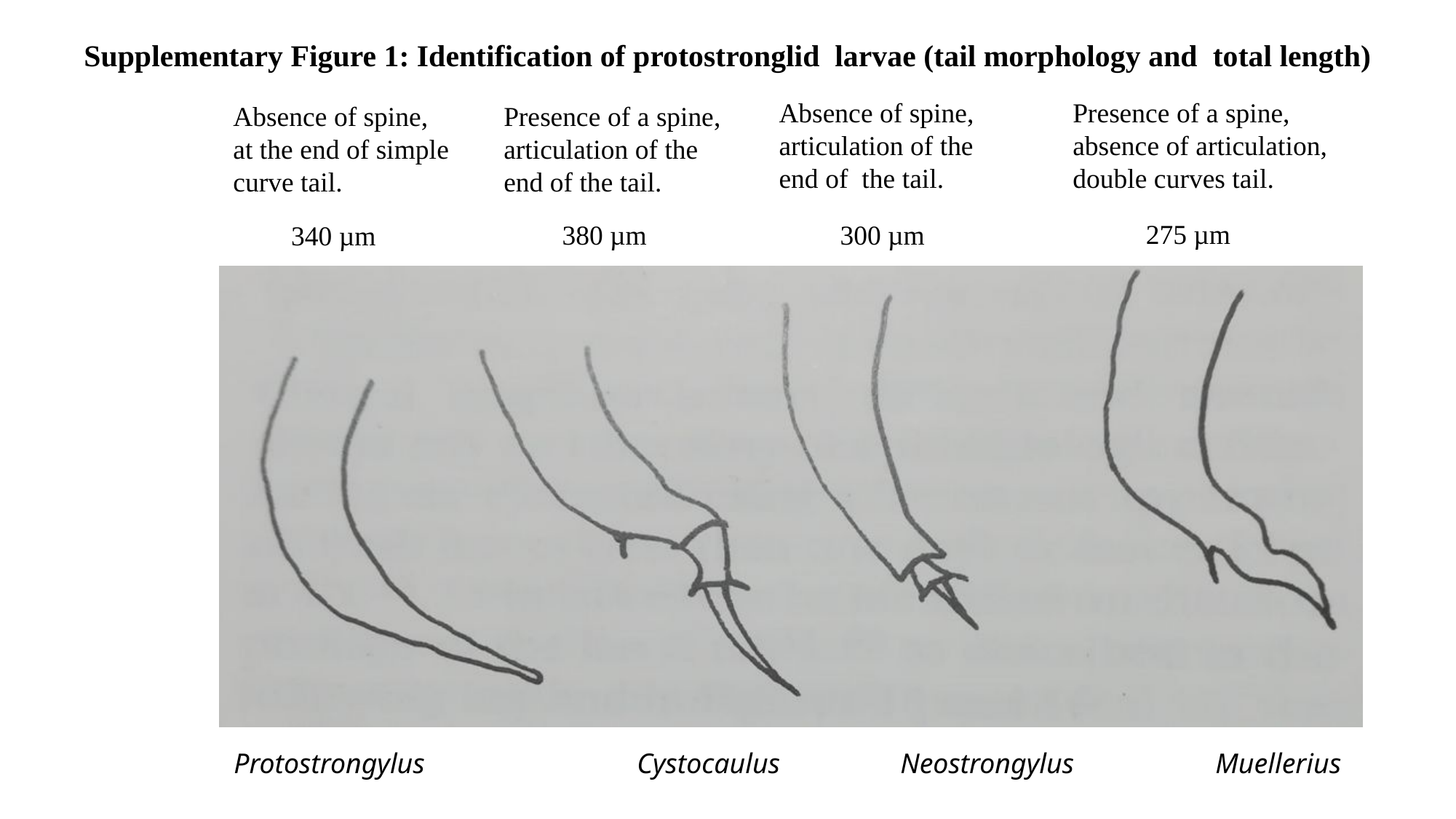

Supplementary Figure 1: Identification of protostronglid larvae (tail morphology and total length)
Absence of spine,
articulation of the
end of the tail.
Presence of a spine,
absence of articulation,
double curves tail.
Absence of spine,
at the end of simple
curve tail.
Presence of a spine,
articulation of the
end of the tail.
275 µm
380 µm 300 µm
340 µm
 Protostrongylus Cystocaulus Neostrongylus Muellerius
